# Factors Influencing Non-Surgical Root Canal Treatment Outcomes in Mandibular Second Molars: A Retrospective Cone-Beam Computed Tomography Analysis

**DOI:** 10.3390/jcm13102931

**Published:** 2024-05-16

**Authors:** Da-Min Park, Woo-Hyun Seok, Ji-Young Yoon

**Affiliations:** 1 Department of Conservative Dentistry, Section of Dentistry, Seoul National University Bundang Hospital, Seongnam 13620, Republic of Korea; 54668@snubh.org; 2 Department of Public Health Administration, Yanggugun Public Health Center, Yanggu-gun 24522, Republic of Korea; whseok96@naver.com

**Keywords:** non-surgical endodontics, periapical diseases, cone-beam computed tomography, treatment outcomes

## Abstract

**Background/Objectives:** This study aimed to investigate the influence of the root canal morphology and various treatment variables on the outcomes of root canal treatments (RCTs) in mandibular second molars, assessed through cone-beam computed tomography (CBCT) imaging. **Methods:** A total of 150 CBCT images were examined, comprising 100 cases of persistent endodontic infections and 50 of previously treated root canals with normal apices in the mandibular second molars. CBCT was utilized to evaluate the root canal configuration, the radiographic quality of coronal restorations and treated canal systems, and the presence of periapical lesions. Statistical analyses were performed to explore the correlations between these factors. **Results:** The presence of a C-shaped root canal configuration did not demonstrate a significant correlation with periapical lesions (*p* = 0.05). Factors influencing endodontic treatment outcomes included missing canals (*p* = 0.018), underfilling or overfilling (*p* = 0.045), and inadequate coronal restoration (*p* = 0.006). Missing a canal was identified as the variable most significantly associated with periapical lesions (OR = 3.103). Inhomogeneous root canal obturation was more commonly observed in C-shaped root canals (*p* < 0.001). **Conclusions:** Regardless of the root canal morphology of mandibular second molars, successful RCT depends on thorough disinfection to eliminate any untreated canals, precise three-dimensional filling of the canals at the correct working length, and a securely sealed coronal restoration to prevent leakage.

## 1. Introduction

Root canal treatment (RCT) hinges on achieving complete sterilization and sealing of the canal interior [1]. To achieve this, it is essential to ensure proper shaping, irrigation, and three-dimensional filling of the canals. The success of each RCT step is significantly impacted by the anatomical features of the canal, with complexities like isthmi and fins known to decrease success rates [2,3]. Beyond the anatomy, intra- and post-operative factors related to the treatment quality are also crucial determinants of RCT success [4]. The presence of missed canals, the state of canal obturation, and the quality of coronal restoration have been widely recognized as crucial factors influencing the success or failure of RCT [5,6].

The root canal morphology of the mandibular second molar is widely recognized for its complexity and variability [7]. Among these variations, a C-shaped canal, where the buccal aspect of the canal systems is fused, is known to have a relatively high occurrence rate [8]. Treating C-shaped canals is difficult due to their complicated anatomical features [9]. Isthmi in C-shaped canals can serve as reservoirs for bacteria and debris, which may not be effectively reached or cleaned using traditional shaping and cleaning techniques [10]. Additionally, three-dimensional sealing of the root canal is challenging, making the mandibular second molar one of the most difficult teeth for RCT. Persistent endodontic infection leads to the failure of RCT [11]. The American Association of Endodontists (AAE) Case Difficulty Assessment Standards indeed categorize teeth with a complex canal morphology, such as C-shaped canals or severe curvature, as high difficulty cases. Additionally, second or third molars are considered highly difficult due to their location, anatomy, and often challenging access. It is suggested that the use of cone-beam computed tomography (CBCT) should be considered to accurately evaluate such difficult cases [12]. 

Numerous studies have aimed to uncover factors contributing to the failure of RCT by investigating the anatomical features, pre-operative pulpal status, and treatment variables of teeth displaying persistent endodontic infection [13]. These investigations aid in comprehending the complexity of RCT and considering various factors to enhance clinical outcomes. Prior research on C-shaped root canals predominantly focused on anatomical features through the analysis of extracted teeth [8,14,15,16]. However, to explore the correlation between post-RCT clinical symptoms and the anatomical structure, CBCT emerges as a valuable tool [6]. CBCT allows for evaluating root canal configurations and treatment quality without the need for tooth extraction [17]. The insights gained from CBCT offer clues that may help us to pinpoint the underlying causes of patient discomfort.

The aim of this study was to assess the influence of the root canal morphology and treatment quality on the outcomes of RCT in mandibular second molars, through a retrospective analysis conducted using CBCT imaging. Additionally, this study examined any potential relationship between C-shaped canals and the quality of RCT.

## 2. Materials and Methods

### 2.1. Case Selection

This study was approved by the Institutional Review Board (IRB no. B-2308-849-103) of the Seoul National University Bundang Hospital. The case group for this study comprised 100 patients who visited the Department of Conservative Dentistry from July 2020 to June 2023 with complaints leading to a consultation for endodontic retreatment in a mandibular second molar that showed periapical radiolucency and caused persistent discomfort. They were diagnosed as either having symptomatic apical periodontitis or a periapical abscess and underwent CBCT for further evaluation with their consent. 

The control group consisted of 50 patients who visited the Department of Oral and Maxillofacial Surgery in a same period. These individuals had undergone CBCT for the extraction of the mandibular third molar and had an adjacent second molar that had previously undergone RCT but had a currently normal periapical status, presenting no clinical symptoms.

- Inclusion criteria: Endodontically treated mandibular molar with its root growth completed, which had to be fully included in CBCT’s imaging range, with clear and complete images available.
- Exclusion criteria: Presence of periodontitis at least in a moderate stage. Diagnosis of conditions other than pulpal or periapical lesions (such as fibro-osseous lesions, benign neoplasms, oral cancer, etc.). Difficulty in radiographic interpretation due to metal artifacts associated with the tooth.

### 2.2. CBCT Analysis

CBCT images were taken with a Kodak 9500 3D system (Carestream Health, Rochester, NY, USA) at a tube voltage of 80 kV, a tube current of 15 mA, and an exposure time of 10.8 s. The resulting images consisted of axial cross-sectional slices with a thickness of 0.2 mm, parallel to the occlusal plane.

**Figure 1:**
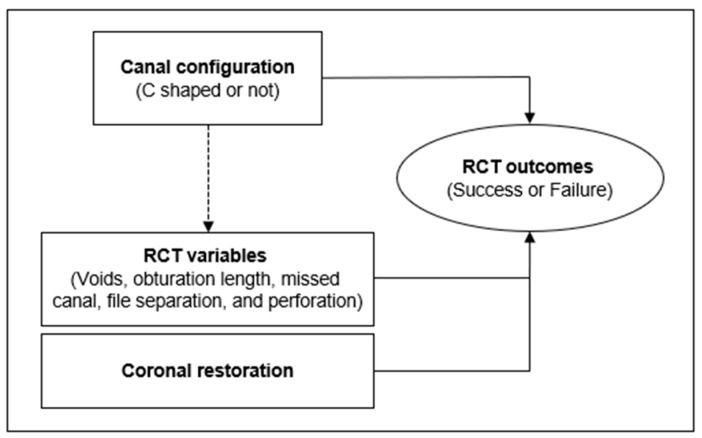
Study design. The impacts of root canal features and the treatment variables on the outcomes of root canal treatment were individually investigated. Additionally, the correlation between a C-shaped canal and the treatment quality was explored.

All images of axial, sagittal, and coronal sliced samples from each scan were analyzed by 2 examiners using the On Demand3D app (Cybermed, Seoul, Korea). Multiple host and treatment variables were recorded from the patients’ records. According to the study design, the following analysis was conducted (Figure 1). 

#### 2.2.1. An Anatomical Factor: Root Canal Morphology Classification

**Figure 2:**
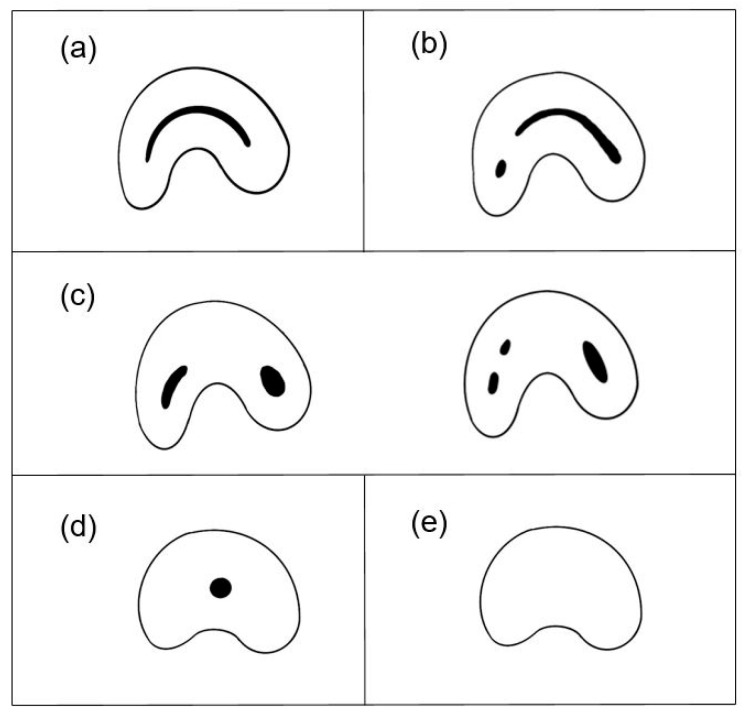
Fan’s classification of C-shaped canals [7]. (**a**) C1, Uninterrupted ‘C’ with no separation or division. (**b**) C2, Canal shape resembles a semicolon. (**c**) C3, Two or three separate canals. (**d**) C4, Only one round or oval canal. (**e**) C5, No canal lumen (usually seen near the apex only).

A C-shaped canal generally occurs when a transverse section of the root canal is in the shape of the letter “C” [8]. The root canal shows various types of shapes as it repeats, dividing into a few branches and reuniting through anastomosis, following the long axis of the root. Usually, a C-shaped canal is not completely connected from the canal orifice to the apical foramen. Therefore, in this study, the C-shaped canal was defined as a tooth showing the aforementioned C shape at least once in the root canal section when it was observed in the axial view of CBCT [9]. The subtypes of C-shaped canals were further classified into C1 to C5 based on the shape observed in the axial view of the middle 1/3 of the root, following the classification proposed by Fan [7] (Figure 2). 

#### 2.2.2. Treatment Factors: Intra- and Post-Operative Factors

The following treatment factors were chosen as criteria for assessing the quality of RCT (Table 1). Obturation density, missed canals, obturation level, and iatrogenic problems were intra-operative factors, while coronal restoration was a post-operative factor.

**Table 1:** Treatment variables examined through CBCT imaging.

Factor	Description	
Obturation density [1]	Good	Homogeneous radiopaque material and no visible space. No more than 2 small voids (<1 mm)
	Poor	Non-uniform radiodensity, with the canal space visible laterally and apically. Isthmus area that had not been treated (Figure 3a)
Missed canals [18]	Unfilled canals appearing from cemento-enamel junction to apex including canals splitting from a main canal at coronal, mid, or apical third (Figure 3b)	
Obturation level [1]	Obturation level of a filling material was measured in millimeters relative to the radiographic apex	
	Normal	Fillings within 2 mm short of the radiographic apex
	Abnormal	Fillings more than 2 mm short of the radiographic apex. Excess root filling. Sealer extrusion
Iatrogenic problems	File separation. Perforation (present/absent) (Figure 3c,d)	
Coronal restoration [18]	Adequate	Any permanent restoration that appeared intact radiographically and had no comment on the clinical examination record
	Inadequate	Any permanent restoration with detectable radiographic signs of overhangs, open margins, or recurrent caries or comments such as “ill-fitting margin” or “secondary dental caries” on the clinical examination record

### 2.3. Statistical Analysis

Chi-squared tests were applied to examine the relationship between the outcome of RCT and the canal shape of the mandibular second molar. Additionally, multiple logistic regression analysis was conducted to assess how anatomical and treatment factors influenced the failure of root canal treatment. A significance level (*p*-value) of 0.05 was chosen for all analyses. Statistical analyses were performed using SPSS ver28.0 (IBM Corporation, Armonk, NY, USA).

## 3. Results

### 3.1. An Anatomical Factor: The Root Canal Morphology

**Figure 4:**
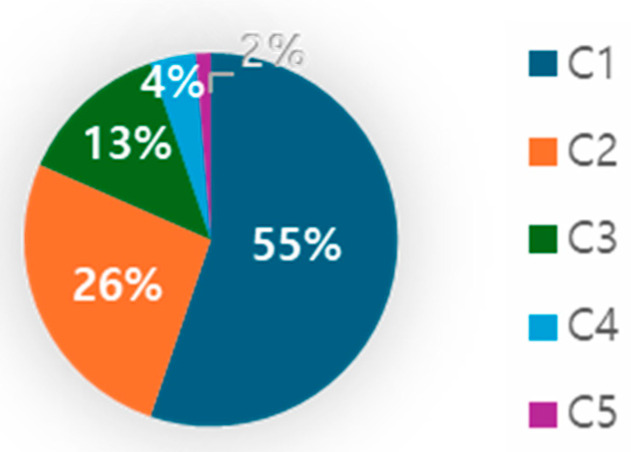
The subtype distribution of the C-shaped canal.

To assess the anatomical complexity of the root canal, we evaluated whether it exhibited a C-shaped configuration or not, and we examined the impact of this configuration on the success rate of root canal treatment. As can be seen in Table 2, the results from the examination using CBCT revealed that out of the total 150 teeth, 77 (51.3%) had a C-shaped canal configuration. The success rate for C-type canals was 26%, compared to 41.1% for non-C-type canals. The C-shaped canal configuration did not significantly influence the success rate of root canal treatment (*p* = 0.05). Among the subtypes of C-shaped canals, C1 (uninterrupted ‘C’ with no separation or division) was the most prevalent, accounting for 55% of the total, followed by C2 (canal shape resembling a semicolon), which constituted 26% (Figure 4).

### 3.2. Intra- and Post-Operative Treatment Factors: Missed Canals, Obturation Length, Obturation Density, Iatrogenic Problems, and Coronal Restoration

We investigated the influences of missed canals, obturation length, inadequate obturation density, iatrogenic events, and coronal leakage on the failure of root canal treatment (Table 3). In cases of missed or untreated canals, the failure rate was 82.6%, whereas it was 59.6% when all canals were treated. Analysis of the obturation length’s correlation with the success rate showed a lower success rate of 14.3% with an inappropriate length, compared to 41.1% with an appropriate length. The presence of voids in the root canal on CBCT imaging, resulting in inhomogeneous root canal obturation, led to a failure rate of 77.5%, compared to 54.3% with an adequately filled canal. Iatrogenic problems like file separation or perforation were detected in nine teeth. The coronal restoration status was analyzed in 146 teeth, excluding those with previously removed restorations. The failure rate of RCT was higher, at 78.9%, when coronal restoration was inappropriate, compared to 53.3% with well-done restoration. As can be seen in Table 4, according to our logistic regression analysis, a missed canal, improper obturation length, and coronal leakage were significant variables influencing the success or failure of root canal treatment. If there was a missed canal, the likelihood of endodontic failure was 3.103 times higher, an improper obturation length increased the failure rate by 2.909 times, and the presence of coronal leakage raised it by 3.057 times. According to these findings, the presence of a missed canal emerges as the most significant factor influencing the failure of RCT.

### 3.3. The Correlation between an Anatomical Factor and Treatment Factors

To investigate the influence of the anatomical canal morphology on the quality of endodontic treatment, the treatment factors were compared according to canal configurations. The incidence of an inhomogeneous obturation density was significantly higher in C-type canals compared to non-C-type canals (*p* < 0.001). There was no significant difference in the degrees of prevalence of missed canals, inadequate obturation lengths, or iatrogenic problems between C-type and non-C-type canals (Table 5).

## 4. Discussion

When identifying the causes of RCT failure, it is essential to consider both anatomical factors and treatment factors. The root canal complexity increases the difficulty of RCT and makes proper canal cleaning and filling more challenging. As a result, any remaining bacteria and their biofilms can lead to the failure of RCT and subsequently progress to apical periodontitis or an apical abscess [19,20]. The mandibular second molar is known to have the most complicated canal system of all teeth [21]. Therefore, this study aimed to investigate the impact of anatomical and treatment factors on RCT outcomes for mandibular second molars. 

Whether or not the root canal has a “C shape” is not a significant factor influencing the success or failure of RCT. In another study comparing the healing outcomes of C-shaped mandibular second molars, the success rate for teeth with a “C shape” was 70.9%, while the success rate for teeth without a “C shape” was 66.6%, with no significant difference observed [22]. There are studies suggesting a lower success rate of RCT in mandibular molars [23]. However, there are also studies indicating no significant differences in success rates among teeth. For instance, one study concluded that the success rate of treatment was not adversely affected by the tooth type or anatomical complexity [4]. It is true that the complexity of the root canal morphology can impact the complete disinfection of the root canal. However, the C-shaped form is a part of root canal complexity. In addition to the C shape, other factors determining complexity include lateral canals, apical ramifications, isthmi, curvature, and so on. It is believed that these factors collectively influence the shaping and obturation of the root canal [24,25,26,27].

Other than root canal anatomy, when considering the effect of the treatment quality on the outcomes of RCT, the obturation level and the presence of a missed canal and coronal leakage act as significant factors. The working length and obturation level are known to significantly affect the results of RCT. It is reported that if canal fillings are 2 mm short of the root apex, then the success rate of RCT drops to 68~77%, and if the canal is overfilled through the apex, then the RCT shows about a 75% success rate [1,28]. Likewise, the present study also showed a significant decrease in the success rate of RCT if the root canal was over- or underfilled. In addition, missed canals emerged as a significant factor contributing to the failure of RCT in this study, as well. In cases where a canal was missed, the failure rate of RCT was 82.6%. This aligns with the findings of previous studies that examined the impact of missed canals on periapical lesions [6]. From the etiological point of view, it seems reasonable that an infected and untreated root canal could trigger an apical lesion. Accordingly, many case studies assert that a missed canal has a close relationship with an apical lesion, with odds ratios ranging from about 4.4 to 6.25 [6,17,29].

On the other hand, the obturation density and iatrogenic problems were not identified as significant factors influencing the outcome of RCT in this study. There are conflicting findings about the effect of intracanal instrument fracture on the prognosis of RCT [30,31]. In this regard, McGuigan [32] suggests that the outcome of a fractured instrument depends on the presence of apical disease before RCT. However, since there was no available information on the apical status before RCT in this study, it was challenging to analyze the relationship between instrument fracture and the success or failure of RCT. In addition, the low incidence of iatrogenic problems (such as file separation and perforation) in this study posed a challenge when attempting to accurately assess their correlation with the success rate of root canal treatment. In terms of the obturation density, it was previously found that the quality of the coronal restoration played a more significant role in determining the endodontic success than the quality of the root filling [33,34]. These results are consistent with our study’s findings, which suggested that coronal leakage significantly influenced the failure of root canal treatment.

When examining differences in treatment factors based on the canal configuration, we found that a C-shaped morphology may significantly affect the attainment of an inhomogeneous obturation density. C-shaped canals are characterized by canals interconnected by fins and isthmuses, making it challenging to achieve three-dimensional sealing of the root canal system with conventional endodontic materials. Therefore, in cases of C-shaped canals, it is necessary to consider various techniques for achieving three-dimensional canal filling after adequate debridement and irrigation have been completed [35]. As can be seen in Table 4, missing a canal is the most significant factor contributing to endodontic failure. Among the 38 cases where endodontic treatment failed due to the presence of a missed canal, there were 22 cases with C-shaped canals and 16 cases with non-C-shaped canals. In eight cases where RCT was successful despite the presence of a missed canal, three had C-shaped canals and five had non-C-shaped canals. Therefore, it can be inferred that the presence of a missed canal is the most crucial factor for succeeding in RCT, which is more important than whether or not the canal is C-shaped.

In this study, 51.3% of the subjects exhibited a C-shaped canal in the mandibular second molar. Previous studies have reported that the occurrence of a C-shaped canal ranges widely from 3 to 39.2% worldwide and is significantly more common in Asian populations than elsewhere. In South Korea, in particular, the C-shaped canal occurrence rate in the mandibular second molar is reported to be around 30 to 40% [36]. The higher prevalence of C-shaped root canals in the present investigation compared to other studies is presumably attributable to the patient groups, composed of individuals referred from local dental clinics due to root canal treatment failure. Among the subtypes of C-shaped canals, C1, which refers to an uninterrupted C-shaped root canal, was the most common, aligning with the findings from previous research [37,38]. The canal subtype made no significant difference in terms of the occurrence of persistent endodontic infection.

This study analyzed CBCT images of root-canal-treated teeth. After the introduction of computed tomography (CT) into endodontics by Tachibana and Matsumoto in 1990, CBCT, capable of capturing images with low radiation, has been used to assess the anatomical form and pathological elements of root canals [39]. CBCT enables three-dimensional cross-sectional evaluation without the need for tooth extraction [40]. In a study comparing the accuracies of various radiographic methods, CBCT demonstrated the highest accuracy in determining the root canal configuration. In terms of accuracy, it is comparable with the modified canal staining and clearing technique performed on extracted teeth [41]. Consequently, in endodontic practice, it serves as a valuable diagnostic aid for formulating treatment plans or assessing treatment outcomes, especially when significant anatomical deviations in root canal anatomy are suspected from 2D images [42]. However, it is not feasible to perform CBCT for every endodontic treatment. In cases where patients continue to complain of discomfort after RCT or when anomalies or pathological issues within the root canal are suspected based on 2D radiographs, CBCT imaging should be considered.

Studies report that the success rate of primary root canal treatment ranges from 92% to 96% when there is no presence of apical periodontitis before the procedure. However, if apical periodontitis is present, the success rate drops to 62–83% [43]. Hence, it is hard to separate the success or failure of RCT from the apical status before the treatment. However, this study focused on teeth for which RCT had already been completed. A limitation was that the pre-operative condition of the pulp and apex of the targeted teeth was not included. Additionally, this study only analyzed the canal morphology in terms of whether or not it was C-shaped, excluding other morphological factors such as extreme curvature or the presence of additional roots like radix entomolaris or radix paramolaris [44]. That exclusion could be considered a limitation of this study, as well. Additional research is proposed to explore other anatomical variations not covered in the current study. This will provide a more comprehensive understanding of the complexities of the root canal morphology and help refine endodontic treatment approaches to address a wider range of anatomical variations encountered in clinical practice.

In conclusion, based on our evaluation of the impact of the root canal morphology and treatment quality on persistent endodontic infection using CBCT imaging, we can surmise that successful RCT for mandibular second molars necessitates thorough disinfection to address any untreated canals, precise three-dimensional canal obturation at the correct working length, and a securely sealed coronal restoration to prevent leakage, irrespective of the root canal morphology. Using dental loupes or a microscope, identifying canal variations with 3D images, maintaining a proper apical seal, and implementing appropriate coronal restoration using high-quality materials and adequate isolation are suggested clinically as endodontic strategies to improve the overall success of treatments.

## Figures and Tables

**Figure 3:**
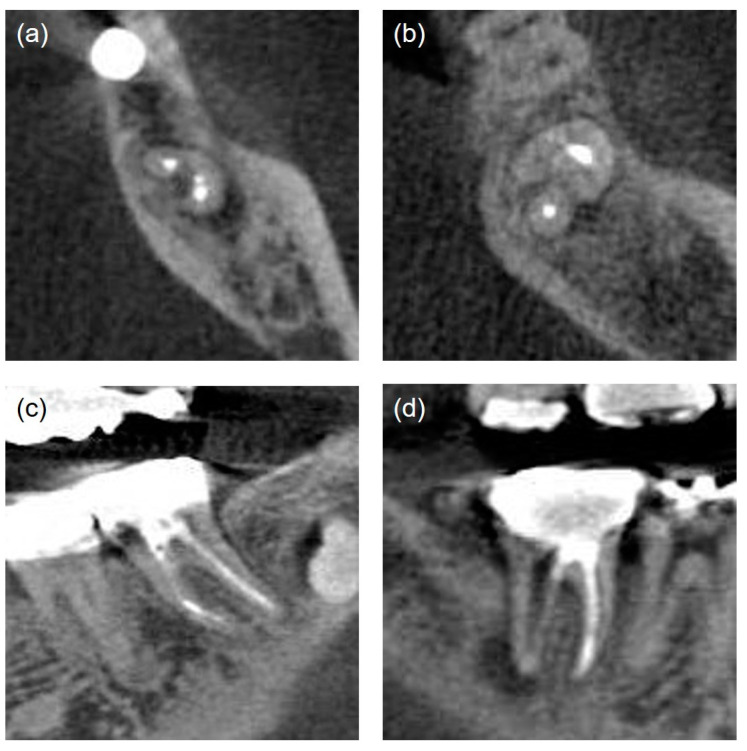
The quality of root canal treatment was evaluated by analyzing CBCT cross-sectional images. (**a**) Untreated isthmus area of the C-shaped canal in the mandibular right second molar, axial view. (**b**) Missed mesio-lingual canal in the mandibular right second molar, axial view. (**c**) Instrument fracture in the mesial root canal of the mandibular left second molar, coronal view. (**d**) Perforation in the furcation area of the mandibular right second molar, coronal view.

**Table 2:** Endodontic treatment outcome relative to anatomical factor.

	C-Shaped N = 77 (100%)	Non C-Shaped N = 73 (100%)	Total N = 150
Success	20 (26%)	30 (41.1%)	50
Failure	57 (74%)	43 (58.9%)	100
x^2^(*p*)	3.856 (*p* = 0.05)		

Significant *p*-value at 0.05 level.

**Table 3:** Endodontic treatment outcome according to treatment factors.

Treatment Factor	N	Endodontic Outcome	
		Success (N = 50)	Failure (N = 100)
Missed canal			
Present	46	8 (17.4%)	38 (82.6%)
Absent	104	42 (40.4%)	62 (59.6%)
Obturation length			
Adequate	107	44 (41.1%)	63 (58.9%)
Inadequate	42	6 (14.3%)	36 (85.7%)
Obturation density			
Poor	80	18 (22.5%)	62 (77.5%)
Good	70	32 (45.7%)	38 (54.3%)
Iatrogenic problem			
Present	9	3 (33.3%)	6 (66.7%)
Absent	141	47 (33.3%)	94 (66.7%)
Coronal restoration			
Adequate	75	35 (46.7%)	40 (53.3%)
Inadequate	71	15 (21.1%)	56 (78.9%)

**Table 4:** Endodontic treatment outcome according to anatomical and treatment factors. Logistic regression analysis.

	Endodontic Failure				
	B	S.E.	Wald	*p*	Exp (B)
C configuration	0.115	0.504	0.053	0.819	1.122
Missed canal	1.132	0.478	5.606	0.018 *	3.103
Obturation length	1.068	0.533	4.019	0.045 *	2.909
Obturation density	0.572	0.508	1.267	0.260	1.772
Iatrogenic events	−0.110	0.819	0.018	0.893	0.896
Coronal leakage	1.117	0.405	7.619	0.006 *	3.057

B, coefficient for the constant; S.E., standard error; Exp (B), exponentiation of the B constant, which is an odds ratio. * Significant difference (*p* < 0.05).

**Table 5:** Treatment quality according to canal configuration.

	C-Shaped	Non C-Shaped	X^2^	*p*-Value
Missed canal (N = 46)	25 (54.3%)	21 (45.7%)	0.241	0.623
Inadequate obturation length (N = 42)	26 (61.9%)	16 (38.1%)	2.450	0.118
Poor obturation density (N = 80)	65 (81.3%)	15 (18.7%)	61.416	<0.001 *
Iatrogenic problem (N = 9)	3 (33.3%)	6 (66.7%)		0.318 ^†^

* Significant *p*-value at 0.05 level. ^†^ Fisher’s exact test.

## Data Availability

The data supporting the reported results are not provided due to ethical restrictions, but can be provided upon request.

## References

[1] Chugal N.M., Clive J.M., Spangberg L.S. (2003). Endodontic infection: Some biologic and treatment factors associated with outcome. Oral. Surg. Oral. Med. Oral. Pathol. Oral. Radiol. Endod..

[2] Vertucci F.J. (2005). Root canal morphology and its relationship to endodontic procedures. Endod. Top..

[3] Lloyd A., Uhles J.P., Clement D.J., Garcia-Godoy F. (2014). Elimination of intracanal tissue and debris through a novel laser-activated system assessed using high-resolution micro-computed tomography: A pilot study. J. Endod..

[4] Ng Y.L., Mann V., Rahbaran S., Lewsey J., Gulabivala K. (2008). Outcome of primary root canal treatment: Systematic review of the literature -- Part 2. Influence of clinical factors. Int. Endod. J..

[5] Tronstad L., Asbjornsen K., Doving L., Pedersen I., Eriksen H.M. (2000). Influence of coronal restorations on the periapical health of endodontically treated teeth. Endod. Dent. Traumatol..

[6] Baruwa A.O., Martins J.N.R., Meirinhos J., Pereira B., Gouveia J., Quaresma S.A., Monroe A., Ginjeira A. (2020). The Influence of Missed Canals on the Prevalence of Periapical Lesions in Endodontically Treated Teeth: A Cross-sectional Study. J. Endod..

[7] Fan B., Cheung G.S., Fan M., Gutmann J.L., Bian Z. (2004). C-shaped canal system in mandibular second molars: Part I—Anatomical features. J. Endod..

[8] Melton D.C., Krell K.V., Fuller M.W. (1991). Anatomical and histological features of C-shaped canals in mandibular second molars. J. Endod..

[9] Kato A., Ziegler A., Higuchi N., Nakata K., Nakamura H., Ohno N. (2014). Aetiology, incidence and morphology of the C-shaped root canal system and its impact on clinical endodontics. Int. Endod. J..

[10] Grocholewicz K., Lipski M., Weyna E. (2009). Endodontic and prosthetic treatment of teeth with C-shaped root canals. Ann. Acad. Med. Stetin..

[11] Nair P.N. (2006). On the causes of persistent apical periodontitis: A review. Int. Endod. J..

[12] AAE Endodontic Case Difficulty Assessment Form and Guidelines. https://www.aae.org/specialty/wp-content/uploads/sites/2/2022/01/CaseDifficultyAssessmentFormFINAL2022.pdf.

[13] Ng Y.L., Mann V., Rahbaran S., Lewsey J., Gulabivala K. (2007). Outcome of primary root canal treatment: Systematic review of the literature—Part 1. Effects of study characteristics on probability of success. Int. Endod. J..

[14] Kotoku K. (1985). Morphological studies on the roots of Japanese mandibular second molars. Shikwa Gakuho.

[15] Carlsen O. (1990). Root complex and root canal system: A correlation analysis using one-rooted mandibular second molars. Scand. J. Dent. Res..

[16] Fan W., Fan B., Gutmann J.L., Cheung G.S. (2007). Identification of C-shaped canal in mandibular second molars. Part I: Radiographic and anatomical features revealed by intraradicular contrast medium. J. Endod..

[17] Karabucak B., Bunes A., Chehoud C., Kohli M.R., Setzer F. (2016). Prevalence of Apical Periodontitis in Endodontically Treated Premolars and Molars with Untreated Canal: A Cone-beam Computed Tomography Study. J. Endod..

[18] Song M., Park M., Lee C.Y., Kim E. (2014). Periapical status related to the quality of coronal restorations and root fillings in a Korean population. J. Endod..

[19] Siqueira Junior J.F., Rocas I.D.N., Marceliano-Alves M.F., Perez A.R., Ricucci D. (2018). Unprepared root canal surface areas: Causes, clinical implications, and therapeutic strategies. Braz. Oral. Res..

[20] Jara C.M., Hartmann R.C., Bottcher D.E., Souza T.S., Gomes M.S., Figueiredo J.A.P. (2018). Influence of apical enlargement on the repair of apical periodontitis in rats. Int. Endod. J..

[21] Maggiore C., Gallottini L., Resi J.P. (1998). Mandibular first and second molar. The variability of roots and root canal system. Minerva Stomatol..

[22] Ahn H.R., Moon Y.M., Hong S.O., Seo M.S. (2016). Healing outcomes of root canal treatment for C-shaped mandibular second molars: A retrospective analysis. Restor. Dent. Endod..

[23] Barbakow F.H., Cleaton-Jones P., Friedman D. (1980). An evaluation of 566 cases of root canal therapy in general dental practice. 2. Postoperative observations. J. Endod..

[24] Siqueira J.F., Rocas I.N., Lopes H.P. (2002). Patterns of microbial colonization in primary root canal infections. Oral. Surg. Oral. Med. Oral. Pathol. Oral. Radiol. Endod..

[25] Ricucci D., Siqueira J.F. (2010). Biofilms and apical periodontitis: Study of prevalence and association with clinical and histopathologic findings. J. Endod..

[26] Ricucci D., Siqueira J.F., Bate A.L., Pitt Ford T.R. (2009). Histologic investigation of root canal-treated teeth with apical periodontitis: A retrospective study from twenty-four patients. J. Endod..

[27] Versiani M.A., Martins J., Ordinola-Zapata R. (2023). Anatomical complexities affecting root canal preparation: A narrative review. Aust. Dent. J..

[28] Lin L.M., Skribner J.E., Gaengler P. (1992). Factors associated with endodontic treatment failures. J. Endod..

[29] Costa F., Pacheco-Yanes J., Siqueira J.F., Oliveira A.C.S., Gazzaneo I., Amorim C.A., Santos P.H.B., Alves F.R.F. (2019). Association between missed canals and apical periodontitis. Int. Endod. J..

[30] Spili P., Parashos P., Messer H.H. (2005). The impact of instrument fracture on outcome of endodontic treatment. J. Endod..

[31] Crump M.C., Natkin E. (1970). Relationship of broken root canal instruments to endodontic case prognosis: A clinical investigation. J. Am. Dent. Assoc..

[32] McGuigan M.B., Louca C., Duncan H.F. (2013). The impact of fractured endodontic instruments on treatment outcome. Br. Dent. J..

[33] Ray H.A., Trope M. (1995). Periapical status of endodontically treated teeth in relation to the technical quality of the root filling and the coronal restoration. Int. Endod. J..

[34] Jang Y.E., Kim Y., Kim S.Y., Kim B.S. (2024). Predicting early endodontic treatment failure following primary root canal treatment. BMC Oral. Health.

[35] Walid N. (2000). The use of two pluggers for the obturation of an uncommon C-shaped canal. J. Endod..

[36] Jin G.C., Lee S.J., Roh B.D. (2006). Anatomical study of C-shaped canals in mandibular second molars by analysis of computed tomography. J. Endod..

[37] Jafarzadeh H., Wu Y.N. (2007). The C-shaped root canal configuration: A review. J. Endod..

[38] Fernandes M., de Ataide I., Wagle R. (2014). C-shaped root canal configuration: A review of literature. J. Conserv. Dent..

[39] Patel S., Durack C., Abella F., Shemesh H., Roig M., Lemberg K. (2015). Cone beam computed tomography in Endodontics—A review. Int. Endod. J..

[40] Kim Y., Lee S.J., Woo J. (2012). Morphology of maxillary first and second molars analyzed by cone-beam computed tomography in a korean population: Variations in the number of roots and canals and the incidence of fusion. J. Endod..

[41] Neelakantan P., Subbarao C., Subbarao C.V. (2010). Comparative evaluation of modified canal staining and clearing technique, cone-beam computed tomography, peripheral quantitative computed tomography, spiral computed tomography, and plain and contrast medium-enhanced digital radiography in studying root canal morphology. J. Endod..

[42] Chan F., Brown L.F., Parashos P. (2023). CBCT in contemporary endodontics. Aust. Dent. J..

[43] de Chevigny C., Dao T.T., Basrani B.R., Marquis V., Farzaneh M., Abitbol S., Friedman S. (2008). Treatment outcome in endodontics: The Toronto study--phase 4: Initial treatment. J. Endod..

[44] Calberson F.L., De Moor R.J., Deroose C.A. (2007). The radix entomolaris and paramolaris: Clinical approach in endodontics. J. Endod..

